# Nutritional Status of Children Under Two Years of Age in the Devbhumi Dwarka District, Gujarat: A Descriptive Cross-Sectional Study

**DOI:** 10.7759/cureus.27445

**Published:** 2022-07-29

**Authors:** Somen Saha, Apurvakumar Pandya, Devang Raval, Manoj S Patil

**Affiliations:** 1 Public Health, Indian Institute of Public Health, Gandhinagar, IND; 2 School of Epidemiology and Public Health, Jawaharlal Nehru Medical College, Datta Meghe Institute of Medical Sciences, Wardha, IND; 3 Epidemiology, Indian Institute of Public Health, Gandhinagar, IND; 4 Public Health, Parul Institute of Public Health, Parul University, Vadodara, IND; 5 Research and Development, Jawaharlal Nehru Medical College, Datta Meghe Institute of Medical Sciences, Wardha, IND

**Keywords:** children under two, india, gujarat, devbhumi dwarka, nutrition status

## Abstract

Improvements in the analysis of child nutrition status can be helpful in increasing the understanding of the magnitude and critical causes of undernutrition. The present study aimed to assess the nutritional status and related factors in children under two years of age in the Devbhumi Dwarka District of Gujarat, India. A descriptive cross-sectional study was conducted for which the sample size was calculated using Open Epi and considering a 20% non-response rate. The sample size for the study was 1200, but the achieved sample size was 1301. Statistical analysis was performed to identify significant determinants of under-nutrition separately for stunting, wasting, and underweight. The prevalence of wasting, underweight, and stunting was 14%, 17%, and 32%, respectively. Prevalence of low birth weight was recorded as 14% in the district. The overall prevalence of overweight was 20% and 6% as per weight for height and weight for age criteria, respectively. The proportion of children with exclusive breastfeeding was observed to be decreasing with increasing age from birth to six months. About 70% of children were exclusively breastfed at the age of six months. Chi-square analyses revealed that parity and spacing are significant determinants of under-nutrition in children under two years in the Devbhumi Dwarka District. A multipronged and convergence approach is needed to combat the menace of child malnutrition.

## Introduction

Malnutrition is a serious public health challenge in India. According to the World Health Organization (WHO), malnutrition refers to deficiencies, excesses, or imbalances in a person's energy intake and nutrients [1]. Types of malnutrition are undernutrition, overnutrition, specific deficiency diseases, and nutritional imbalance. High malnutrition rates among children are alarming in India [2-4]. The prevalence of underweight and wasted children was higher in Gujarat compared to India's national average [2,5,6]. It is well acknowledged that undernutrition has a negative impact on the population's productivity and is a major contributor to the global disease burden, including maternal and child mortality. Moreover, malnutrition is associated with increased health care costs, reduced productivity, and slow economic growth, which perpetuates the cycle of poverty and ill health [7].

Some children are undernourished in the first two years of life. But if they gain weight rapidly later, they are reported to show a high risk of chronic nutrition-related diseases [7]. The nutritional interventions during this period manifest the greatest impact on child health, survival, and development [8]. Several studies explored the prevalence and described associated risk factors of undernutrition among children under five in India [9,10] and other developing countries [11,12]. But there is a lack of literature reporting the prevalence and determinants of malnutrition in children under two years of age. The present study was conducted to assess the prevalence of malnutrition and to identify the factors related to it among children below two years of age in the Devbhumi Dwarka District of Gujarat.

## Materials and methods

Study design

A descriptive cross-sectional study was conducted from February to March of 2020. The present study was conducted in four blocks: Khambhaliya, Bhanvad, Kalyanpur, and Dwarka blocks of the Devbhumi Dwarka District in Gujarat.

Sample size calculation

The sample size was calculated using Open Epi software [13]. Considering a 20% non-response rate, with a design effect of 1 at 99.99% significance level, and the prevalence assuming 50%, the calculated sample size was 1,292. The same size was calculated using the formula: sample size n=(DEFF*Np(1-p))/((d2/Z21-α/2*(N-1)+p*(1-p)), where p is prevalence. We achieved a 1,301 sample size for the study. The sample size calculation followed the principles used in the WHO survey [14]. 

Operational definitions

The following operational definitions were used for analysis: low birth weight is defined as less than 2500 gm [8]. Prevalence of underweight (low weight-for-age), stunting (low height-for-age), wasting (low weight-for-height), and overweight (high weight-for-height) was evaluated by using of z-score cut-off points (i.e. SD scores); < -2SD, < -3SD, and > +2SD [15,16]. Exclusive breastfeeding is a proportion of infants 0-6 months of age who received no oral food or fluid intake except breast milk and oral rehydration solution drops or syrups containing vitamins, minerals, or medicines [15,17].

Data collection tool

Based on the references and recommendations of previous national surveys in India [5,6] and the published literature on child malnutrition [9,10,15], the survey tool was developed. Respondents, including key informants (mother of the respective child), participated in the study. Demographic and socioeconomic details and variables of child and maternal history were sought by interviewing and reviewing medical records. Anthropometry measurements, i.e., height and weight, were recorded using standard validated instruments, wooden infant stadiometer, and digital weighing scales. Z-scores of all anthropometric data were calculated as per the WHO growth standard by using WHO Anthro Analyzer (version 1, 2018) software. After the training of field investigators on survey tools, interview methods, and anthropometric measurements, a standardization exercise was conducted with 20 participants to assess the technical error of measurement. 

Data analysis

Data was entered in Microsoft Office Excel (Microsoft, Redmond, Washington), and analysis was carried out in the SPSS version 20 statistics software (IBM Inc., Armonk, New York) [17]. The continuous variables were presented using frequency and percentage. For categorical variables, the Chi-square test of independence was performed to identify a significant association between various factors and malnutrition. 

Ethical considerations

Informed consent was sought from each key informant (mother) of the study, and formal approval was obtained from state and district authorities. Ethical approval was obtained from the Institutional Ethics Committee of the Indian Institute of Public Health, Gandhinagar. Procedures to assure confidentiality of data and participating individuals were strictly observed.

## Results

Maternal History

Mothers of 1,301 children from the Khambhaliya, Bhanvad, Kalyanpur, and Dwarka blocks of the Devbhumi Dwarka District of Gujarat were interviewed in the study. All respondents of the present study were mothers of children assessed for malnutrition. Of all respondents, 53.2% were aged 25 to 34 years. Only 4% of respondents were aged more than 35 years. The majority of respondents (66.9%) were from General Caste, while 20.6% were from socially and economically backward class, 9.1% and 3.4% were from Schedule Tribe and Schedule Caste, respectively. Around one-fifth (19.1%) of respondents were Muslims, while the majority (80.9%) were Hindu. Out of all respondents, 88.7% were homemakers while 11.3% were dealing with other activities, like labor, service sector, or self-employment. Only 9.3% of respondents had reported an average monthly family income of more than ±20,000 rupees, while 55.5% had reported less than 10,000 rupees. The details of sociodemographic features are given in Table 1.

**Table 1:** Sociodemographic profile of participants ST - Scheduled Tribe,  SC - Scheduled Caste, SEBC - socially and educationally backward classes, OBC - Other Backward Class, SES - socioeconomic status

Age (N=1297)	N	%
18 to 25	555	42.8%
25 to 34	690	53.2%
35 to 44	49	3.8%
45 and above	3	0.2%
Caste (N=1258)	N	%
ST	115	9.1%
SC	43	3.4%
SEBC/ OBC	259	20.6%
General	841	66.9%
Religion (N=1301)	N	%
Hindu	1053	80.9%
Muslim	248	19.1%
Occupation (N=1301)	N	%
Housewife	1154	88.7%
Laborer	120	9.2%
Service	17	1.3%
Self-employed	10	0.8%
SES (N=1241)	N	%
< 5000	162	13.1%
5000 - 10000	526	42.4%
10000 - 15000	266	21.4%
15000 - 20000	172	13.9%
>20000	115	9.3%
Literacy (N=1301)	N	%
Illiterate	352	27.1%
Primary	619	47.6%
Secondary	229	17.6%
Higher secondary	35	2.7%
Graduate and above	66	5.1%
Parity (N=1301)	N	%
≤ 2	991	76.2%
> 2	310	23.8%
Spacing between two pregnancies (N=799)	N	%
≤ 3 years	326	40.8%
> 3 years	473	59.2%

Among all respondents, overall literacy was 72.9%. It was observed that around 56.8% of women reported to be multiparous, and among them, 37% of mothers had spacing less than two years between two pregnancies. The spacing between two children remains between one to eight years. Data reveals a higher rate of institutional delivery (99%), with 49% of deliveries conducted in public health facilities. The majority of babies (80%) were delivered normally.

Profile of children assessed

A total of 1301 children under the age of 24 months were assessed in the study. Of them, 35% and 65% belonged to ages less than six months and seven to 24 months, respectively. The proportion of male (52.3%) children was higher than females (47.7%). The overall prevalence of low birth weight was reported at 14%. The details are given in Table 2.

**Table 2:** Profile of children assessed during the study

Age	N (%)
0-6 Months Child	452 (35%)
7-24 Months Child	849 (65%)
Sex	N (%)
Male	681 (52.3%)
Female	620 (47.7%)
Low birth weight (<2.5 kg)	176 (14%)

Breastfeeding practice among children up to six months

In 246 (55.2%) babies, breastfeeding was initiated during the first hour of birth, and about 22.4% of newborns were breastfed after 24 hours of birth. At the time of the survey, about 11.4% of respondents were facing difficulties in breastfeeding. Critical difficulties reported were sore nipples, mastitis, breast engorgement, breast abscess, maternal illness, child’s inability to suck, breast refusal, not enough milk, etc. The proportion of children with exclusive breastfeeding decreased with increasing age from birth to 6 months. About 70% of children had received exclusive breastfeeding till the age of six months. 

Difficulties in breastfeeding were the main reported reason behind giving liquid other than breastmilk. The age-specific proportion of children aged 0 to six months who received feed other than breast milk is given in Table 3. The liquids other than milk included plain water, infant formula mix, fresh animal milk, fruit juice, yogurt, and thin porridge. This practice declines in case of mother sickness by 26.5 %. It was also reported that children were provided with top milk (6.3%), oral rehydration solutions (ORS) (2%), and plain water (2%) during illness. Around 15% of respondents reported providing nothing during sickness. The details are given in Table 3.

**Table 3:** Breastfeeding practices for children below six months

Breastfeeding Initiation (N=446)	
Timing of initiation of breastfeeding after birth	N (%)
Within 1 hour after birth	246(55.2)
From 1 to 3 hours after birth	63(14.1)
More than 3 hours after birth	31(7)
After 24 hours	100(22.4)
Difficulties in breastfeeding (N=446)	
Any difficulty during breastfeeding	51(11.4%)
No difficulty	395 (88.6%)
Exclusive breastfeeding (N=446)	
Age in months	
0 (N=25)	21 (84%)
1 (N=52)	43 (82.7%)
2 (N=67)	58 (86.6%)
3 (N=70)	53 (75.7%)
4 (N=70)	54 (77.1 %)
5 (N=112)	85 (75.9%)
6 (N=50)	35 (70.0%)
Breastfeeding while sickness (N=446)	
Breastfeed given during child sickness	328 (73.5%)

Prevalence of malnutrition

During the study, out of all study respondents, about 83% of children’s length and weight could be measured. Among them, the prevalence of wasting, underweight, and stunting was 14%, 17%, and 32%, respectively. The overall prevalence of overweight was 20% and 6% as per weight for height and weight for age criteria, respectively. The nutritional profile of children under two is given in Table 4.

**Table 4:** Nutritional status of children below the age of 24 months (N=1185)

	Normal	Moderate to severe	Overweight
Height for age	811 (68%)	374 (32%)	NA
Weight for height	783 (66%)	169 (14%)	233 (20%)
Weight for age	905 (76%)	204 (17%)	76 (6%)

Factors related to wasting and underweight

Various child, maternal, and environmental factors were analyzed against the status of malnutrition. Table 5 presents descriptive statistics. The prevalence of wasting (< -2 SD) was higher in children less than six months (22.6%) than in elder children (14%). Similarly, overweight (OW) was observed to be higher in this age group. Children under the age of six months had (12%) overweight than older children (5.8%).

**Table 5:** Child factors related to malnutrition

Nutritional indicators	Height for age		Weight for height		Weight for age	
	Stunting	No stunting (> - 2SD)	Wasting	Overweight (> +2SD)	Underweight	Overweight (> +2SD)
Age of child						
Age 0 to 6 months	111 (24.1%)	350 (75.9%)	104 (22.6%)	119 (25.8%)	83 (17.8%)	56 (12.0%)
Age 7 to 24 months	303 (36.6%)	525 (63.4%)	116 (14.0%)	150 (18.1%)	158 (19.0%)	48 (5.8%)
Sex of child						
Male	231 (34.4%)	441 (65.6%)	122 (18.2%)	147 (21.9%)	133 (19.6%)	58 (8.5%)
Female	183 (29.7%)	434 (70.3%)	98 (15.9%)	122 (19.8%)	108 (17.4%)	46 (7.4%)
Birth weight						
Low	85 (48.3%)	91 (51.7%)	54 (30.7%)	28 (15.9%)	79 (44.9%)	4 (2.3%)
Normal	329 (29.6%)	784 (70.4%)	166 (14.9%)	241 (21.7%)	162 (14.4%)	100 (8.9%)

In contrast, the proportion of underweight children was higher among children older than six months (19%) than younger children (17.8%). The difference in nutritional status as per weight for height (WFH) and weight for age (WFA) in different age groups was found to be statistically significant. No significant gender difference was observed in the nutritional status of children. The low birth weight was found to be related to the current status of nutrition in children under the age of two years. The prevalence of wasting (30.7%) and undernutrition (44.9%) was higher among children with low birth weight than in children with normal birth weight.

Maternal factors like parity, birth spacing, and literacy were included in the analysis (Table 6). No significant differences were observed in WFA among children as per the parity of the women. However, the prevalence of underweight was higher in children of women in more than two parities (22.3%) than in others (17.4%). Similarly, the prevalence of underweight was found higher (23%) in a low birth spacing group than in others (17.1%). Low birth spacing (≤3 years) and low parity (≤2) were found to be associated with being underweight. Prevalence of stunting was found significantly higher among women with ≤3 years of spacing between two consecutive pregnancies (23%) than women with more than three years birth spacing (17%).

**Table 6:** Maternal factors related to malnutrition

Nutritional indicators	Height for age		Weight for height		Weight for age	
	Stunting	No stunting (> - 2SD)	Wasting	Overweight (>+2SD)	Underweight	Overweight (>+2SD)
Parity						
≤ 2	302 (30.8%)	678 (69.2%)	172 (17.6%)	202 (20.6%)	172 (17.4%)	85 (8.6%)
>2	112 (36.2%)	197 (63.8%)	48 (15.5%)	67 (21.7%)	69 (22.3%)	19 (6.1%)
Spacing						
≤ 3 years	111 (34.4%)	212 (65.6%)	61 (18.9%)	59 (18.3%)	75 (23.0%)	19 (5.8%)
> 3 years	303 (31.4%)	663 (68.6%)	159 (16.5%)	210 (21.7%)	166 (17.1%)	85 (8.7%)
Mother’s education						
Illiterate	129 (37.1%)	219 (62.9%)	65 (18.7%)	67 (19.3%)	80 (22.8%)	30 (8.5%)
Up-to primary	190 (30.9%)	425 (69.1%)	101 (16.4%)	129 (21.0%)	101 (16.3%)	47 (7.6%)
Secondary and higher education	95 (29.1%)	231 (70.9%)	54 (16.6%)	73 (22.4%)	60 (18.2%)	27 (8.2%)
Exclusive breastfeeding ≤ 3 months						
Yes	3 (7.7%)	36 (92.3%)	44 (25.6%)	48 (27.9%)	44 (25.1%)	12 (6.9%)
No	44 (25.6%)	128 (74.4%)	17 (43.6%)	4 (10.3%)	7 (17.9%)	5 (12.8%)
Exclusive Breastfeeding 4 to 6 months						
Yes	50 (28.9%)	123 (71.1%)	29 (16.8%)	47 (27.2%)	24 (13.8%)	16 (9.2%)
No	15 (26.3%)	42 (73.7%)	13 (22.8%)	19 (33.3%)	9 (15.5%)	10 (17.2%)
Problems in Breastfeeding						
Yes	10 (20.0%)	40 (80.0%)	10 (20.0%)	13 (26.0%)	15 (29.4%)	5 (9.8%)
No	102 (26.1%)	289 (73.9%)	93 (23.8%)	105 (26.9%)	69 (17.5%)	38 (9.6%)
Breastfeeding during illness						
Yes	90 (27.8%)	234 (72.2%)	64 (19.8%)	97 (29.9%)	53 (16.2%)	30 (9.1%)
No	22 (18.8%)	95 (81.2%)	39 (33.3%)	21 (17.9%)	31 (26.3%)	13 (11.0%)

As shown in Table 6, the prevalence of wasting was lower among children with exclusive breastfeeding than among children aged ≤ 3 months. But being overweight was found to be higher in children with exclusive breastfeeding. The difference was found statically significant. In contrast to this, the prevalence of overweight was found to be lower in children aged four to six months. But the difference was not statistically significant. The indirect factor affecting breastfeeding practice, i.e., problems faced for breastfeeding, was found to be unrelated to malnutrition.

The prevalence of stunting was higher in children aged more than six months (36.6%) compared to younger children (24,.1%), and the difference was statistically significant. There was no significant difference in stunting observed among males and females. Stunting was reported more in children with low birth weight (48.3%) than normal birth weight (29.6%). The prevalence of stunting was found more among children with low spacing (34.4%) and high parity (36.2%) than spacing more than three years (31.4%) and parity less than equal to two (30.8%). Still, the difference found was not statistically significant. Literacy among women was found to be related to stunting. The prevalence of stunting was high among children of illiterate (37.1%) than literate women (30.0%). The prevalence of stunting was found to be lower among children with exclusive breastfeeding (7.7%) in ages less than three months than in children who were not exclusively breastfed (74.4%). Exclusive breastfeeding was found to be related to stunting in children aged less than three months. No statistical difference was found in stunting among children aged more than three months.

## Discussion

The assessment of nutritional status and its predictors provides insight into further strategies and policies to combat malnutrition. Malnutrition is one of the major public health problems. In the present study dual burden of malnutrition was observed. The prevalence of wasting, underweight, and stunting is 14%, 17%, and 32%, respectively, in children under two years of age. It is lower than the state and national average [5,6]. A study conducted in the Narmada district in Gujarat [17] reported a higher prevalence of wasting, stunting, and underweight among children under two were 32.2%, 34.5%, and 39.7%, respectively. Other studies conducted in Gujarat found the prevalence of malnutrition among children under five to be higher than in the present study [18-23]. The prevalence of overweight was 20% per weight for height, which is four times higher than reported by Biro et al. They reported a prevalence of 5.5% based on secondary data in 2016 [22]. In the baseline survey, about 14% of children reported low birth weight, which is less than the national and state average [5,6].

In the present study, 55% of mothers had initiated breastfeeding within one hour of live birth, and 78% were found to exclusively breastfeed. Prevalence of exclusive breastfeeding in the Devbhumi Dwarka District was found to be higher than state and national prevalence as per NFHS-4 & 5. According to the Comprehensive National Nutritional Survey (CNNS 2016-18), 59% of children below the age of six months had initiated breastfeeding within the first hour of live birth, and 58% of children below six months were exclusively breastfeeding in India [2]. The present nutritional survey observed practices such as not giving colostrum, too early introduction of weaning, low feeding frequency, and less emphasis on nutritious foods were recorded.

Children’s age and birth weight were associated with malnutrition, while gender was not related to the malnutrition status in the present study. Similar findings were reported by the study conducted in other districts of Gujarat state [18,19]. However, contrasting to the present study, Ahmed et al. [22] evidenced that the male sex was more prone to become malnourished. Moreover, low birth weight was associated with malnutrition in the present study, which was reported in previous studies [20-23].

The present study compared maternal factor-like parity, birth spacing, and literacy between normal and malnourished children. Among them, maternal literacy was found to be related to stunting, and spacing was found to be related to undernutrition. The prevalence of malnutrition was higher in respondents who had lower or no education and where the spacing between two consecutive birth was less than three years. The parity of the respondent was found not related to malnutrition. Similar to the present study, some other studies [24-27] found maternal factors, literacy, and birth spacing associated with malnutrition in children. Breastfeeding during sickness was found to be related to malnutrition. The association of breastfeeding with malnutrition was not associated with malnutrition [20], but sub-group analysis in the present study revealed that exclusive breastfeeding at an age less than three months was associated with malnutrition compared to exclusive breastfeeding up to six months.

In summary, the child’s age and maternal factors, like the interval between two consecutive pregnancies and maternal literacy, were associated with undernutrition in children below two years of age. Though, as compared to some recent studies, some behavioral aspects, for example, early initiation of breastfeeding and weaning practices, are better in the Devbhumi Dwarka District, these are the area that still needs to be addressed because the rate of exclusive breastfeeding decreased with the increase of child’s age up to six months. Hence, the role of primary care physicians in nutrition counseling lactating women and monitoring infant and young child feeding practices during post-natal care visits by Auxiliary Nurse Midwife and Accredited Social Health Activists remains crucial.

Child malnutrition is one of the public health challenges. Present study findings throw light on the determinants and magnitude of the nutrient gaps among children under two years of age. Findings can be useful to local administrators and program managers for re-strategizing current interventions, strengthening post-natal care services under the Department of Health and Family Welfare, and implementing integrated child development services under the Department of Women and Child Development. Multipronged and convergence approaches such as integrating family planning education, nutrition literacy, proper infant and child feeding practices, appropriate maternal care, growth monitoring, and nutritional supplementation should be enhanced to combat the menace of child malnutrition.

Potential limitations of this survey-based cross-sectional study include self-reporting bias. Various societal factors are known to influence nutritional status, including social customs, economic status, and the status of the public distribution system. Studying the influence of all such factors on the overall nutritional status of children remains an area of future research.

## Conclusions

The present study underscores the dual burden of malnutrition in the Devbhumi Dwarka District. However, it was noted that the prevalence of wasting, underweight, and stunting was lower in the present study than in the previous state and national surveys. Age of child and maternal factors like the interval between two consecutive pregnancies and maternal literacy was found to be associated with undernutrition in children below two years of age.
